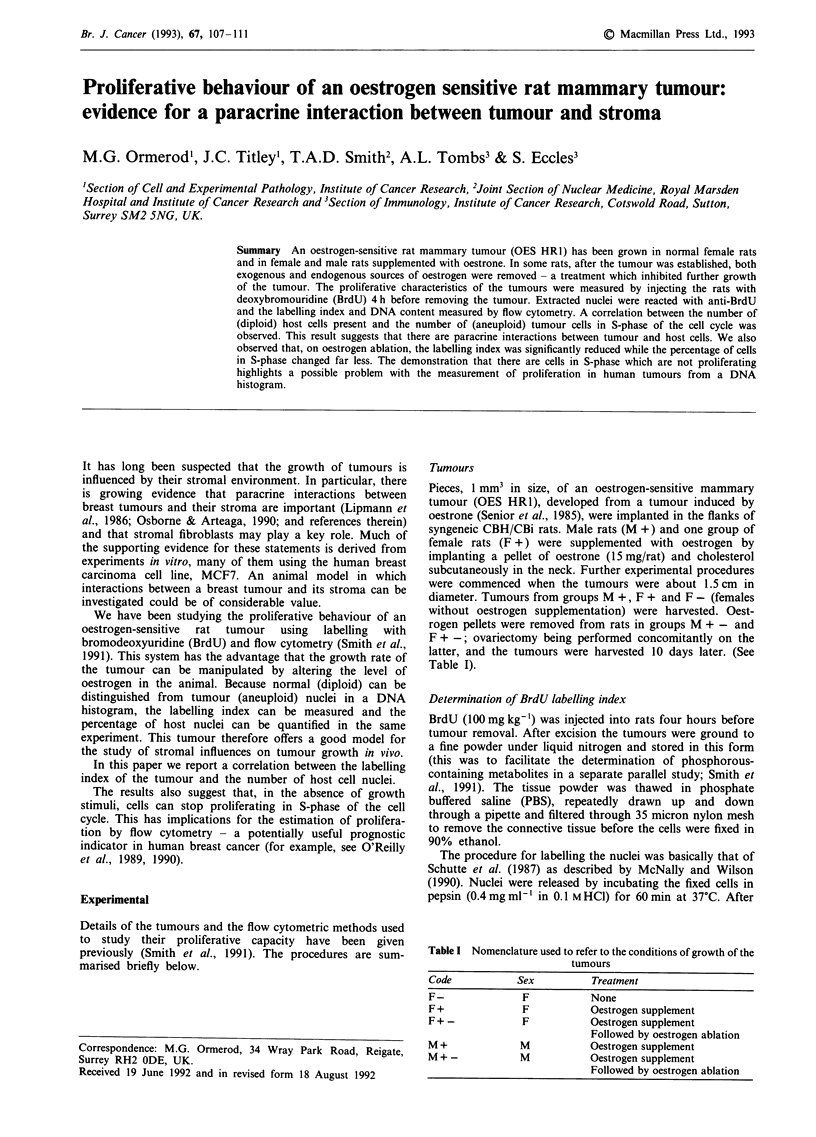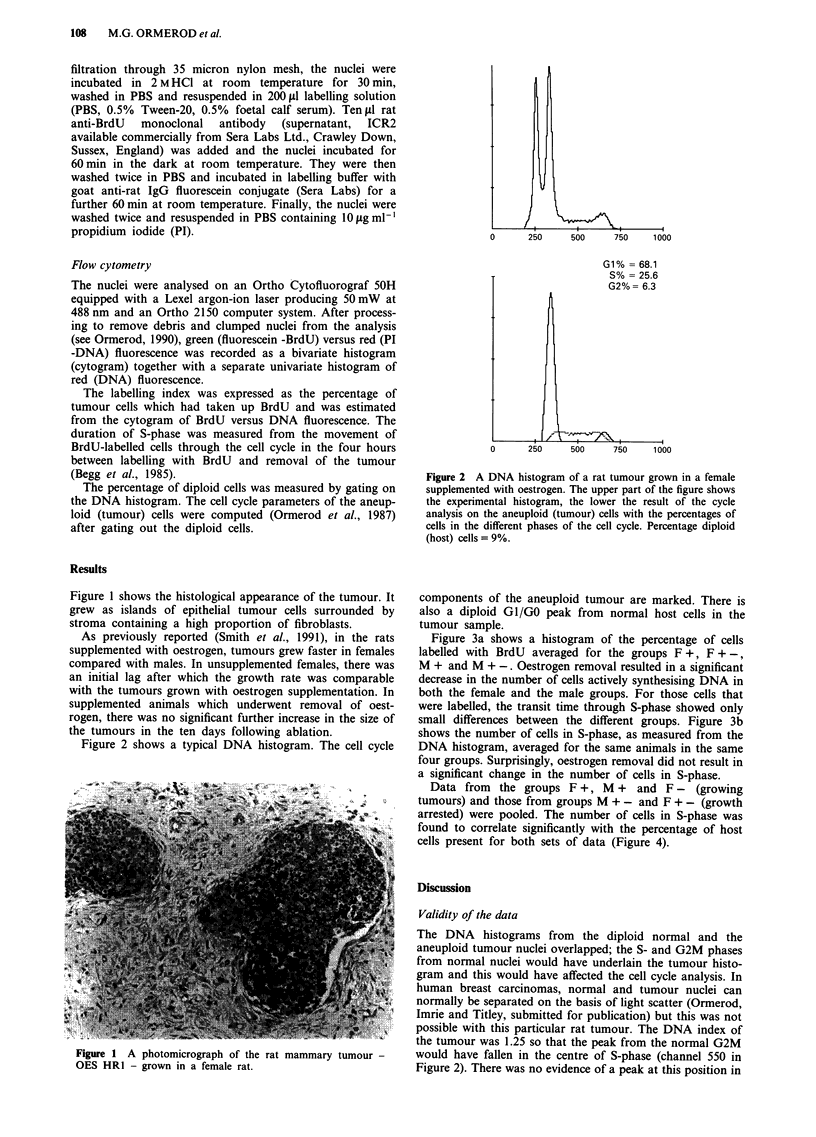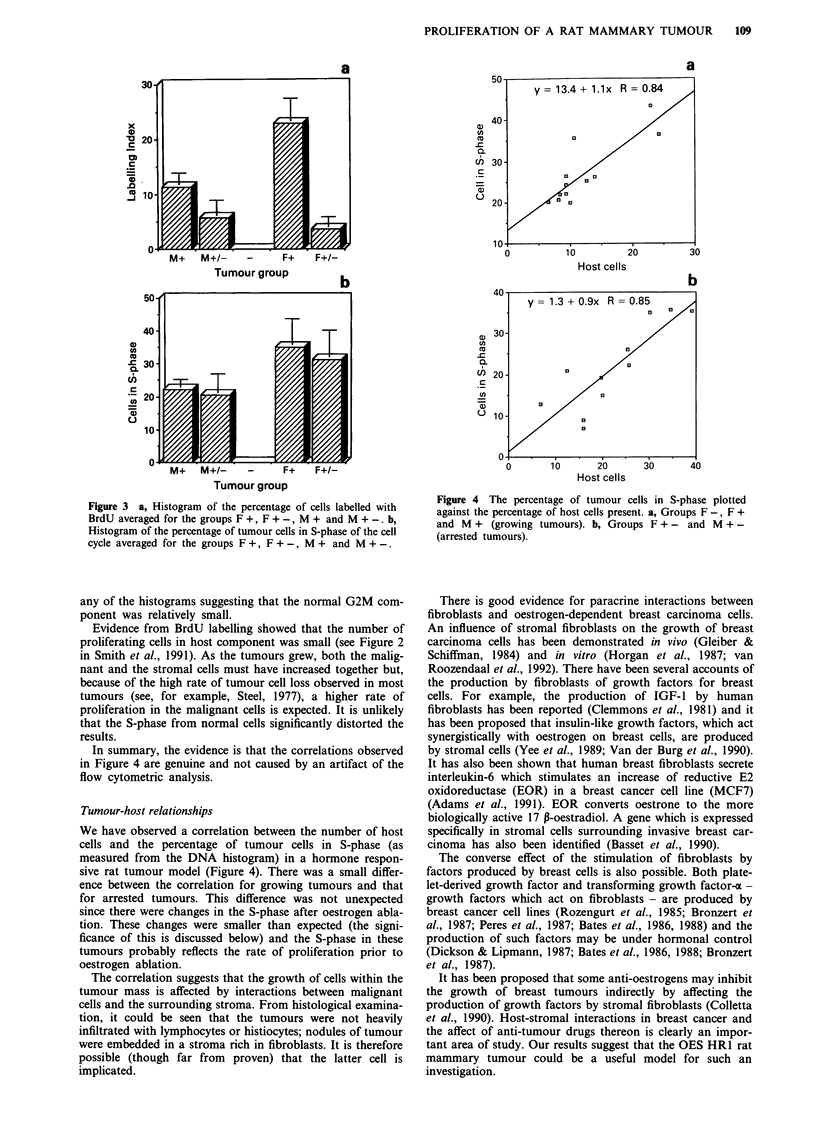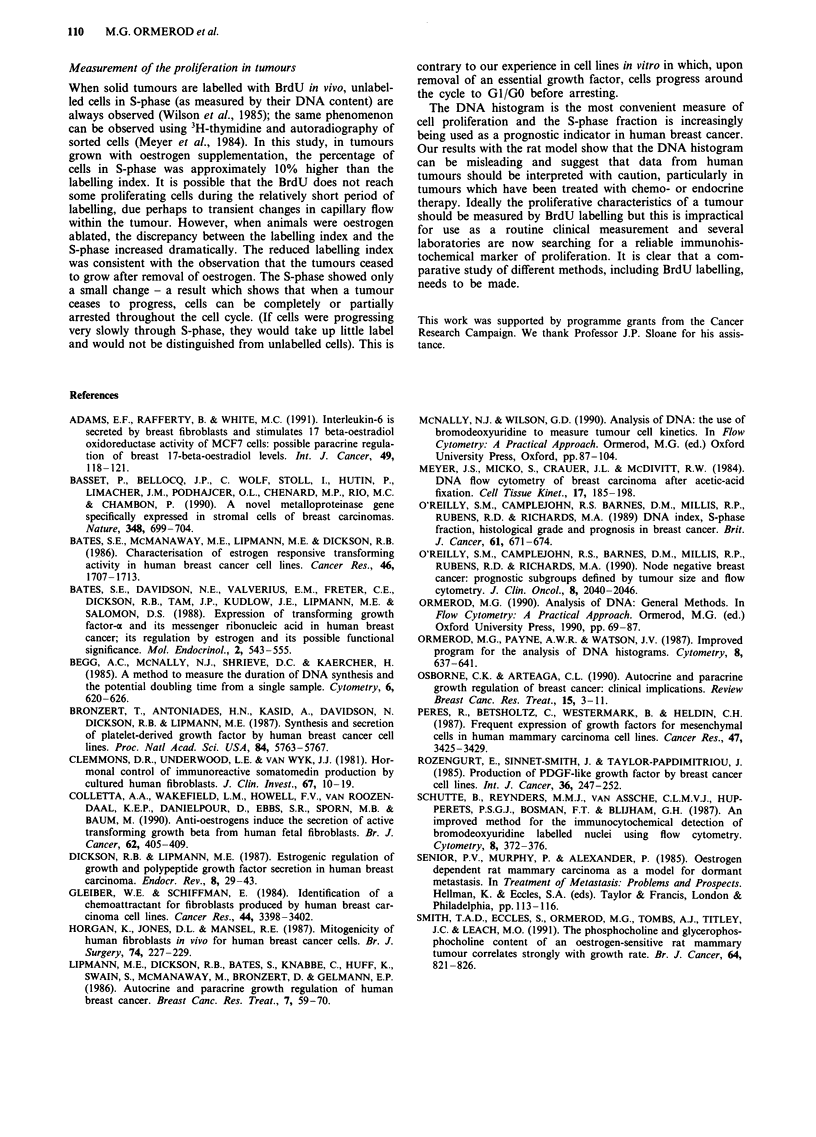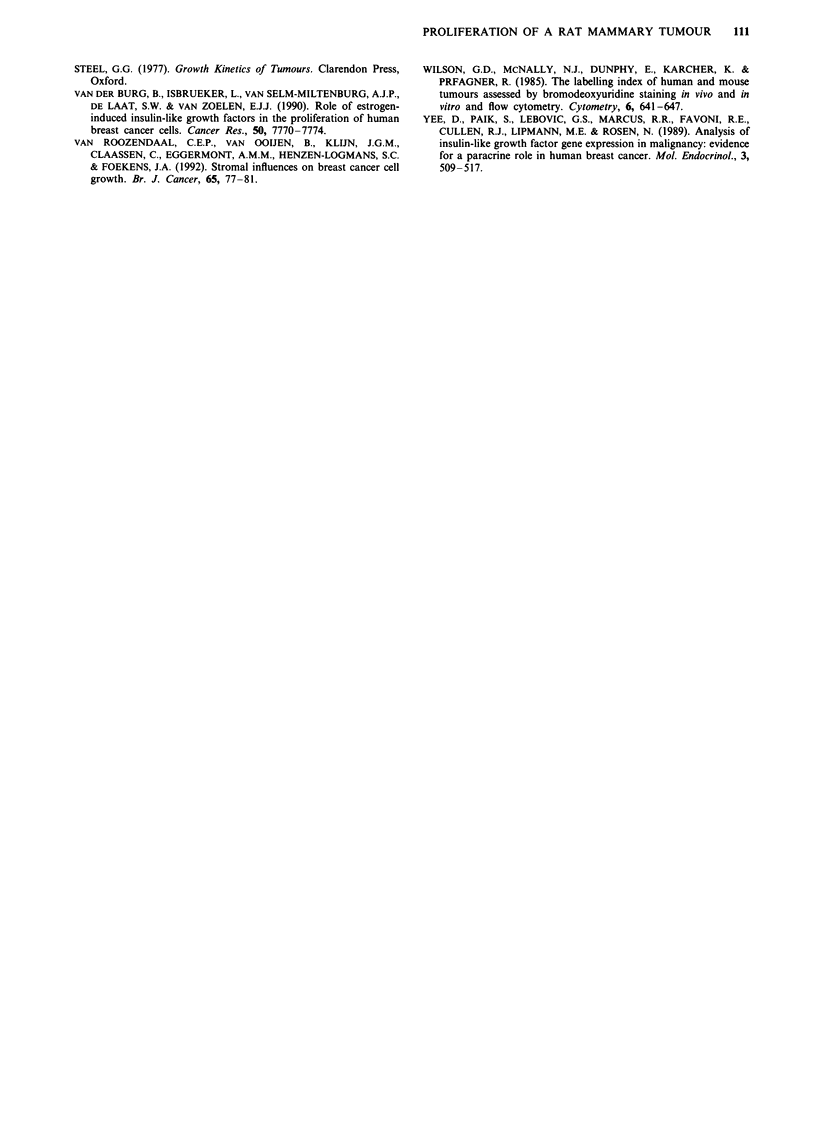# Proliferative behaviour of an oestrogen sensitive rat mammary tumour: evidence for a paracrine interaction between tumour and stroma.

**DOI:** 10.1038/bjc.1993.18

**Published:** 1993-01

**Authors:** M. G. Ormerod, J. C. Titley, T. A. Smith, A. L. Tombs, S. Eccles

**Affiliations:** Section of Cell and Experimental Pathology, Institute of Cancer Research, Sutton, Surrey, UK.

## Abstract

**Images:**


*Br. J. Cancer* (1993), **67**, 107–111 © Macmillan Press Ltd., 1993

### Proliferative behaviour of an oestrogen sensitive rat mammary tumour: evidence for a paracrine interaction between tumour and stroma

M.G. Ormerod[1], J.C. Titley[1], T.A.D. Smith[2], A.L. Tombs[3] & S. Eccles[3]

[1]*Section of Cell and Experimental Pathology, Institute of Cancer Research,* [2]*Joint Section of Nuclear Medicine, Royal Marsden Hospital and Institute of Cancer Research and* [3]*Section of Immunology, Institute of Cancer Research, Cotswold Road, Sutton, Surrey SM2 5NG, UK.*

**Summary** An oestrogen-sensitive rat mammary tumour (OES HR1) has been grown in normal female rats and in female and male rats supplemented with oestrone. In some rats, after the tumour was established, both exogenous and endogenous sources of oestrogen were removed – a treatment which inhibited further growth of the tumour. The proliferative characteristics of the tumours were measured by injecting the rats with deoxybromouridine (BrdU) 4 h before removing the tumour. Extracted nuclei were reacted with anti-BrdU and the labelling index and DNA content measured by flow cytometry. A correlation between the number of (diploid) host cells present and the number of (aneuploid) tumour cells in S-phase of the cell cycle was observed. This result suggests that there are paracrine interactions between tumour and host cells. We also observed that, on oestrogen ablation, the labelling index was significantly reduced while the percentage of cells in S-phase changed far less. The demonstration that there are cells in S-phase which are not proliferating highlights a possible problem with the measurement of proliferation in human tumours from a DNA histogram.

It has long been suspected that the growth of tumours is influenced by their stromal environment. In particular, there is growing evidence that paracrine interactions between breast tumours and their stroma are important (Lipmann *et al.*, 1986; Osborne & Arteaga, 1990; and references therein) and that stromal fibroblasts may play a key role. Much of the supporting evidence for these statements is derived from experiments *in vitro*, many of them using the human breast carcinoma cell line, MCF7. An animal model in which interactions between a breast tumour and its stroma can be investigated could be of considerable value.

We have been studying the proliferative behaviour of an oestrogen-sensitive rat tumour using labelling with bromodeoxyuridine (BrdU) and flow cytometry (Smith *et al.*, 1991). This system has the advantage that the growth rate of the tumour can be manipulated by altering the level of oestrogen in the animal. Because normal (diploid) can be distinguished from tumour (aneuploid) nuclei in a DNA histogram, the labelling index can be measured and the percentage of host nuclei can be quantified in the same experiment. This tumour therefore offers a good model for the study of stromal influences on tumour growth *in vivo*.

In this paper we report a correlation between the labelling index of the tumour and the number of host cell nuclei.

The results also suggest that, in the absence of growth stimuli, cells can stop proliferating in S-phase of the cell cycle. This has implications for the estimation of proliferation by flow cytometry – a potentially useful prognostic indicator in human breast cancer (for example, see O'Reilly *et al.*, 1989, 1990).

### Experimental

Details of the tumours and the flow cytometric methods used to study their proliferative capacity have been given previously (Smith *et al.*, 1991). The procedures are summarised briefly below.

Correspondence: M.G. Ormerod, 34 Wray Park Road, Reigate, Surrey RH2 0DE, UK.
Received 19 June 1992 and in revised form 18 August 1992

### *Tumours*

Pieces, 1 $mm^3$ in size, of an oestrogen-sensitive mammary tumour (OES HR1), developed from a tumour induced by oestrone (Senior *et al.*, 1985), were implanted in the flanks of syngeneic CBH/CBi rats. Male rats (M +) and one group of female rats (F +) were supplemented with oestrogen by implanting a pellet of oestrone (15 mg/rat) and cholesterol subcutaneously in the neck. Further experimental procedures were commenced when the tumours were about 1.5 cm in diameter. Tumours from groups M +, F + and F – (females without oestrogen supplementation) were harvested. Oestrogen pellets were removed from rats in groups M + – and F + –; ovariectomy being performed concomitantly on the latter, and the tumours were harvested 10 days later. (See Table I).

### *Determination of BrdU labelling index*

BrdU (100 mg $kg^{-1}$) was injected into rats four hours before tumour removal. After excision the tumours were ground to a fine powder under liquid nitrogen and stored in this form (this was to facilitate the determination of phosphorous-containing metabolites in a separate parallel study; Smith *et al.*, 1991). The tissue powder was thawed in phosphate buffered saline (PBS), repeatedly drawn up and down through a pipette and filtered through 35 micron nylon mesh to remove the connective tissue before the cells were fixed in 90% ethanol.

The procedure for labelling the nuclei was basically that of Schutte *et al.* (1987) as described by McNally and Wilson (1990). Nuclei were released by incubating the fixed cells in pepsin (0.4 mg $ml^{-1}$ in 0.1 M HCl) for 60 min at 37°C. After

**Table I** Nomenclature used to refer to the conditions of growth of the tumours

| *Code* | *Sex* | *Treatment* |
|---|---|---|
| F– | F | None |
| F+ | F | Oestrogen supplement |
| F+– | F | Oestrogen supplement<br>Followed by oestrogen ablation |
| M+ | M | Oestrogen supplement |
| M+– | M | Oestrogen supplement<br>Followed by oestrogen ablation |

filtration through 35 micron nylon mesh, the nuclei were incubated in 2 M HCl at room temperature for 30 min, washed in PBS and resuspended in 200 µl labelling solution (PBS, 0.5% Tween-20, 0.5% foetal calf serum). Ten µl rat anti-BrdU monoclonal antibody (supernatant, ICR2 available commercially from Sera Labs Ltd., Crawley Down, Sussex, England) was added and the nuclei incubated for 60 min in the dark at room temperature. They were then washed twice in PBS and incubated in labelling buffer with goat anti-rat IgG fluorescein conjugate (Sera Labs) for a further 60 min at room temperature. Finally, the nuclei were washed twice and resuspended in PBS containing 10 $\mu g\ ml^{-1}$ propidium iodide (PI).

*Flow cytometry*

The nuclei were analysed on an Ortho Cytofluorograf 50H equipped with a Lexel argon-ion laser producing 50 mW at 488 nm and an Ortho 2150 computer system. After processing to remove debris and clumped nuclei from the analysis (see Ormerod, 1990), green (fluorescein -BrdU) versus red (PI -DNA) fluorescence was recorded as a bivariate histogram (cytogram) together with a separate univariate histogram of red (DNA) fluorescence.

The labelling index was expressed as the percentage of tumour cells which had taken up BrdU and was estimated from the cytogram of BrdU versus DNA fluorescence. The duration of S-phase was measured from the movement of BrdU-labelled cells through the cell cycle in the four hours between labelling with BrdU and removal of the tumour (Begg et al., 1985).

The percentage of diploid cells was measured by gating on the DNA histogram. The cell cycle parameters of the aneuploid (tumour) cells were computed (Ormerod et al., 1987) after gating out the diploid cells.

**Figure 2** A DNA histogram of a rat tumour grown in a female supplemented with oestrogen. The upper part of the figure shows the experimental histogram, the lower the result of the cycle analysis on the aneuploid (tumour) cells with the percentages of cells in the different phases of the cell cycle. Percentage diploid (host) cells = 9%.

### Results

Figure 1 shows the histological appearance of the tumour. It grew as islands of epithelial tumour cells surrounded by stroma containing a high proportion of fibroblasts.

As previously reported (Smith et al., 1991), in the rats supplemented with oestrogen, tumours grew faster in females compared with males. In unsupplemented females, there was an initial lag after which the growth rate was comparable with the tumours grown with oestrogen supplementation. In supplemented animals which underwent removal of oestrogen, there was no significant further increase in the size of the tumours in the ten days following ablation.

Figure 2 shows a typical DNA histogram. The cell cycle components of the aneuploid tumour are marked. There is also a diploid G1/G0 peak from normal host cells in the tumour sample.

**Figure 1** A photomicrograph of the rat mammary tumour – OES HR1 – grown in a female rat.

Figure 3a shows a histogram of the percentage of cells labelled with BrdU averaged for the groups F+, F+−, M+ and M+−. Oestrogen removal resulted in a significant decrease in the number of cells actively synthesising DNA in both the female and the male groups. For those cells that were labelled, the transit time through S-phase showed only small differences between the different groups. Figure 3b shows the number of cells in S-phase, as measured from the DNA histogram, averaged for the same animals in the same four groups. Surprisingly, oestrogen removal did not result in a significant change in the number of cells in S-phase.

Data from the groups F+, M+ and F− (growing tumours) and those from groups M+− and F+− (growth arrested) were pooled. The number of cells in S-phase was found to correlate significantly with the percentage of host cells present for both sets of data (Figure 4).

### Discussion

*Validity of the data*

The DNA histograms from the diploid normal and the aneuploid tumour nuclei overlapped; the S- and G2M phases from normal nuclei would have underlain the tumour histogram and this would have affected the cell cycle analysis. In human breast carcinomas, normal and tumour nuclei can normally be separated on the basis of light scatter (Ormerod, Imrie and Titley, submitted for publication) but this was not possible with this particular rat tumour. The DNA index of the tumour was 1.25 so that the peak from the normal G2M would have fallen in the centre of S-phase (channel 550 in Figure 2). There was no evidence of a peak at this position in

**Figure 3** **a**, Histogram of the percentage of cells labelled with BrdU averaged for the groups F +, F + –, M + and M + –. **b**, Histogram of the percentage of tumour cells in S-phase of the cell cycle averaged for the groups F +, F + –, M + and M + –.

**Figure 4** The percentage of tumour cells in S-phase plotted against the percentage of host cells present. **a**, Groups F –, F + and M + (growing tumours). **b**, Groups F + – and M + – (arrested tumours).

any of the histograms suggesting that the normal G2M component was relatively small.

Evidence from BrdU labelling showed that the number of proliferating cells in host component was small (see Figure 2 in Smith *et al.*, 1991). As the tumours grew, both the malignant and the stromal cells must have increased together but, because of the high rate of tumour cell loss observed in most tumours (see, for example, Steel, 1977), a higher rate of proliferation in the malignant cells is expected. It is unlikely that the S-phase from normal cells significantly distorted the results.

In summary, the evidence is that the correlations observed in Figure 4 are genuine and not caused by an artifact of the flow cytometric analysis.

### *Tumour-host relationships*

We have observed a correlation between the number of host cells and the percentage of tumour cells in S-phase (as measured from the DNA histogram) in a hormone responsive rat tumour model (Figure 4). There was a small difference between the correlation for growing tumours and that for arrested tumours. This difference was not unexpected since there were changes in the S-phase after oestrogen ablation. These changes were smaller than expected (the significance of this is discussed below) and the S-phase in these tumours probably reflects the rate of proliferation prior to oestrogen ablation.

The correlation suggests that the growth of cells within the tumour mass is affected by interactions between malignant cells and the surrounding stroma. From histological examination, it could be seen that the tumours were not heavily infiltrated with lymphocytes or histiocytes; nodules of tumour were embedded in a stroma rich in fibroblasts. It is therefore possible (though far from proven) that the latter cell is implicated.

There is good evidence for paracrine interactions between fibroblasts and oestrogen-dependent breast carcinoma cells. An influence of stromal fibroblasts on the growth of breast carcinoma cells has been demonstrated *in vivo* (Gleiber & Schiffman, 1984) and *in vitro* (Horgan *et al.*, 1987; van Roozendaal *et al.*, 1992). There have been several accounts of the production by fibroblasts of growth factors for breast cells. For example, the production of IGF-1 by human fibroblasts has been reported (Clemmons *et al.*, 1981) and it has been proposed that insulin-like growth factors, which act synergistically with oestrogen on breast cells, are produced by stromal cells (Yee *et al.*, 1989; Van der Burg *et al.*, 1990). It has also been shown that human breast fibroblasts secrete interleukin-6 which stimulates an increase of reductive E2 oxidoreductase (EOR) in a breast cancer cell line (MCF7) (Adams *et al.*, 1991). EOR converts oestrone to the more biologically active 17 $\beta$-oestradiol. A gene which is expressed specifically in stromal cells surrounding invasive breast carcinoma has also been identified (Basset *et al.*, 1990).

The converse effect of the stimulation of fibroblasts by factors produced by breast cells is also possible. Both platelet-derived growth factor and transforming growth factor-$\alpha$ – growth factors which act on fibroblasts – are produced by breast cancer cell lines (Rozengurt *et al.*, 1985; Bronzert *et al.*, 1987; Peres *et al.*, 1987; Bates *et al.*, 1986, 1988) and the production of such factors may be under hormonal control (Dickson & Lipmann, 1987; Bates *et al.*, 1986, 1988; Bronzert *et al.*, 1987).

It has been proposed that some anti-oestrogens may inhibit the growth of breast tumours indirectly by affecting the production of growth factors by stromal fibroblasts (Colletta *et al.*, 1990). Host-stromal interactions in breast cancer and the affect of anti-tumour drugs thereon is clearly an important area of study. Our results suggest that the OES HR1 rat mammary tumour could be a useful model for such an investigation.

*Measurement of the proliferation in tumours*

When solid tumours are labelled with BrdU *in vivo*, unlabelled cells in S-phase (as measured by their DNA content) are always observed (Wilson *et al.*, 1985); the same phenomenon can be observed using $^3$H-thymidine and autoradiography of sorted cells (Meyer *et al.*, 1984). In this study, in tumours grown with oestrogen supplementation, the percentage of cells in S-phase was approximately 10% higher than the labelling index. It is possible that the BrdU does not reach some proliferating cells during the relatively short period of labelling, due perhaps to transient changes in capillary flow within the tumour. However, when animals were oestrogen ablated, the discrepancy between the labelling index and the S-phase increased dramatically. The reduced labelling index was consistent with the observation that the tumours ceased to grow after removal of oestrogen. The S-phase showed only a small change – a result which shows that when a tumour ceases to progress, cells can be completely or partially arrested throughout the cell cycle. (If cells were progressing very slowly through S-phase, they would take up little label and would not be distinguished from unlabelled cells). This is contrary to our experience in cell lines *in vitro* in which, upon removal of an essential growth factor, cells progress around the cycle to G1/G0 before arresting.

The DNA histogram is the most convenient measure of cell proliferation and the S-phase fraction is increasingly being used as a prognostic indicator in human breast cancer. Our results with the rat model show that the DNA histogram can be misleading and suggest that data from human tumours should be interpreted with caution, particularly in tumours which have been treated with chemo- or endocrine therapy. Ideally the proliferative characteristics of a tumour should be measured by BrdU labelling but this is impractical for use as a routine clinical measurement and several laboratories are now searching for a reliable immunohistochemical marker of proliferation. It is clear that a comparative study of different methods, including BrdU labelling, needs to be made.

This work was supported by programme grants from the Cancer Research Campaign. We thank Professor J.P. Sloane for his assistance.

**References**

ADAMS, E.F., RAFFERTY, B. & WHITE, M.C. (1991). Interleukin-6 is secreted by breast fibroblasts and stimulates 17 beta-oestradiol oxidoreductase activity of MCF7 cells: possible paracrine regulation of breast 17-beta-oestradiol levels. *Int. J. Cancer*, **49**, 118–121.

BASSET, P., BELLOCQ, J.P., C. WOLF, STOLL, I., HUTIN, P., LIMACHER, J.M., PODHAJCER, O.L., CHENARD, M.P., RIO, M.C. & CHAMBON, P. (1990). A novel metalloproteinase gene specifically expressed in stromal cells of breast carcinomas. *Nature*, **348**, 699–704.

BATES, S.E., MCMANAWAY, M.E., LIPMANN, M.E. & DICKSON, R.B. (1986). Characterisation of estrogen responsive transforming activity in human breast cancer cell lines. *Cancer Res.*, **46**, 1707–1713.

BATES, S.E., DAVIDSON, N.E., VALVERIUS, E.M., FRETER, C.E., DICKSON, R.B., TAM, J.P., KUDLOW, J.E., LIPMANN, M.E. & SALOMON, D.S. (1988). Expression of transforming growth factor-α and its messenger ribonucleic acid in human breast cancer; its regulation by estrogen and its possible functional significance. *Mol. Endocrinol.*, **2**, 543–555.

BEGG, A.C., MCNALLY, N.J., SHRIEVE, D.C. & KAERCHER, H. (1985). A method to measure the duration of DNA synthesis and the potential doubling time from a single sample. *Cytometry*, **6**, 620–626.

BRONZERT, T., ANTONIADES, H.N., KASID, A., DAVIDSON, N. DICKSON, R.B. & LIPMANN, M.E. (1987). Synthesis and secretion of platelet-derived growth factor by human breast cancer cell lines. *Proc. Natl Acad. Sci. USA*, **84**, 5763–5767.

CLEMMONS, D.R., UNDERWOOD, L.E. & VAN WYK, J.J. (1981). Hormonal control of immunoreactive somatomedin production by cultured human fibroblasts. *J. Clin. Invest.*, **67**, 10–19.

COLLETTA, A.A., WAKEFIELD, L.M., HOWELL, F.V., VAN ROOZENDAAL, K.E.P., DANIELPOUR, D., EBBS, S.R., SPORN, M.B. & BAUM, M. (1990). Anti-oestrogens induce the secretion of active transforming growth beta from human fetal fibroblasts. *Br. J. Cancer*, **62**, 405–409.

DICKSON, R.B. & LIPMANN, M.E. (1987). Estrogenic regulation of growth and polypeptide growth factor secretion in human breast carcinoma. *Endocr. Rev.*, **8**, 29–43.

GLEIBER, W.E. & SCHIFFMAN, E. (1984). Identification of a chemoattractant for fibroblasts produced by human breast carcinoma cell lines. *Cancer Res.*, **44**, 3398–3402.

HORGAN, K., JONES, D.L. & MANSEL, R.E. (1987). Mitogenicity of human fibroblasts *in vivo* for human breast cancer cells. *Br. J. Surgery*, **74**, 227–229.

LIPMANN, M.E., DICKSON, R.B., BATES, S., KNABBE, C., HUFF, K., SWAIN, S., MCMANAWAY, M., BRONZERT, D. & GELMANN, E.P. (1986). Autocrine and paracrine growth regulation of human breast cancer. *Breast Canc. Res. Treat.*, **7**, 59–70.

MCNALLY, N.J. & WILSON, G.D. (1990). Analysis of DNA: the use of bromodeoxyuridine to measure tumour cell kinetics. In *Flow Cytometry: A Practical Approach*. Ormerod, M.G. (ed.) Oxford University Press, Oxford, pp.87–104.

MEYER, J.S., MICKO, S., CRAUER, J.L. & MCDIVITT, R.W. (1984). DNA flow cytometry of breast carcinoma after acetic-acid fixation. *Cell Tissue Kinet.*, **17**, 185–198.

O'REILLY, S.M., CAMPLEJOHN, R.S. BARNES, D.M., MILLIS, R.P., RUBENS, R.D. & RICHARDS, M.A. (1989) DNA index, S-phase fraction, histological grade and prognosis in breast cancer. *Brit. J. Cancer*, **61**, 671–674.

O'REILLY, S.M., CAMPLEJOHN, R.S., BARNES, D.M., MILLIS, R.P., RUBENS, R.D. & RICHARDS, M.A. (1990). Node negative breast cancer: prognostic subgroups defined by tumour size and flow cytometry. *J. Clin. Oncol.*, **8**, 2040–2046.

ORMEROD, M.G. (1990). Analysis of DNA: General Methods. In *Flow Cytometry: A Practical Approach*. Ormerod, M.G. (ed.) Oxford University Press, 1990, pp. 69–87.

ORMEROD, M.G., PAYNE, A.W.R. & WATSON, J.V. (1987). Improved program for the analysis of DNA histograms. *Cytometry*, **8**, 637–641.

OSBORNE, C.K. & ARTEAGA, C.L. (1990). Autocrine and paracrine growth regulation of breast cancer: clinical implications. *Review Breast Canc. Res. Treat.*, **15**, 3–11.

PERES, R., BETSHOLTZ, C., WESTERMARK, B. & HELDIN, C.H. (1987). Frequent expression of growth factors for mesenchymal cells in human mammary carcinoma cell lines. *Cancer Res.*, **47**, 3425–3429.

ROZENGURT, E., SINNET-SMITH, J. & TAYLOR-PAPDIMITRIOU, J. (1985). Production of PDGF-like growth factor by breast cancer cell lines. *Int. J. Cancer*, **36**, 247–252.

SCHUTTE, B., REYNDERS, M.M.J., VAN ASSCHE, C.L.M.V.J., HUPPERETS, P.S.G.J., BOSMAN, F.T. & BLIJHAM, G.H. (1987). An improved method for the immunocytochemical detection of bromodeoxyuridine labelled nuclei using flow cytometry. *Cytometry*, **8**, 372–376.

SENIOR, P.V., MURPHY, P. & ALEXANDER, P. (1985). Oestrogen dependent rat mammary carcinoma as a model for dormant metastasis. In *Treatment of Metastasis: Problems and Prospects*. Hellman, K. & Eccles, S.A. (eds). Taylor & Francis, London & Philadelphia, pp.113–116.

SMITH, T.A.D., ECCLES, S., ORMEROD, M.G., TOMBS, A.J., TITLEY, J.C. & LEACH, M.O. (1991). The phosphocholine and glycerophosphocholine content of an oestrogen-sensitive rat mammary tumour correlates strongly with growth rate. *Br. J. Cancer*, **64**, 821–826.

STEEL, G.G. (1977). *Growth Kinetics of Tumours*. Clarendon Press, Oxford.

VAN DER BURG, B., ISBRUEKER, L., VAN SELM-MILTENBURG, A.J.P., DE LAAT, S.W. & VAN ZOELEN, E.J.J. (1990). Role of estrogen-induced insulin-like growth factors in the proliferation of human breast cancer cells. *Cancer Res.*, **50,** 7770–7774.

VAN ROOZENDAAL, C.E.P., VAN OOIJEN, B., KLIJN, J.G.M., CLAASSEN, C., EGGERMONT, A.M.M., HENZEN-LOGMANS, S.C. & FOEKENS, J.A. (1992). Stromal influences on breast cancer cell growth. *Br. J. Cancer*, **65,** 77–81.

WILSON, G.D., MCNALLY, N.J., DUNPHY, E., KARCHER, K. & PRFAGNER, R. (1985). The labelling index of human and mouse tumours assessed by bromodeoxyuridine staining *in vivo* and *in vitro* and flow cytometry. *Cytometry*, **6,** 641–647.

YEE, D., PAIK, S., LEBOVIC, G.S., MARCUS, R.R., FAVONI, R.E., CULLEN, R.J., LIPMANN, M.E. & ROSEN, N. (1989). Analysis of insulin-like growth factor gene expression in malignancy: evidence for a paracrine role in human breast cancer. *Mol. Endocrinol.*, **3,** 509–517.